# Supplementary material for: Modulation of Multispecific Transporters by Uncaria tomentosa Extract and Its Major Phytoconstituents
Source: Pharmaceutics. 2024 Oct 25;16(11):1363. doi: 10.3390/pharmaceutics16111363 (PMC11597817; doi:10.3390/pharmaceutics16111363)
Supplement: Supplementary file 1 [file pharmaceutics-16-01363-s001.zip › pharmaceutics-3227303-supplementary.pdf]

Supplementary information

Modulation of multispecific transporters by Uncaria tomentosa and its major phytoconstituents

Nóra Szilvásy[1], Panna Lajer[1], Attila Horváth[2], Katalin Veres[2], Judit Hohmann[2,3], Zsuzsanna Schelz[4], Renáta Minorics[4], István Zupkó[4], Zsuzsanna Gáborik[1], Emese Kis[1], Csilla Temesszentandrási-Ambrus[1]

[1]Charles River Laboratories Hungary, Irinyi J Street 4-20, 1117 Budapest, Hungary

[2]Institute of Pharmacognosy, University of Szeged, Eötvös Street 6, 6720 Szeged, Hungary

[3]ELKH-USZ Biologically Active Natural Products Research Group, University of Szeged, 6720 Szeged, Hungary

[4]Institute of Pharmacodynamics and Biopharmacy, University of Szeged, Eötvös Str. 6, 6720 Szeged Hungary

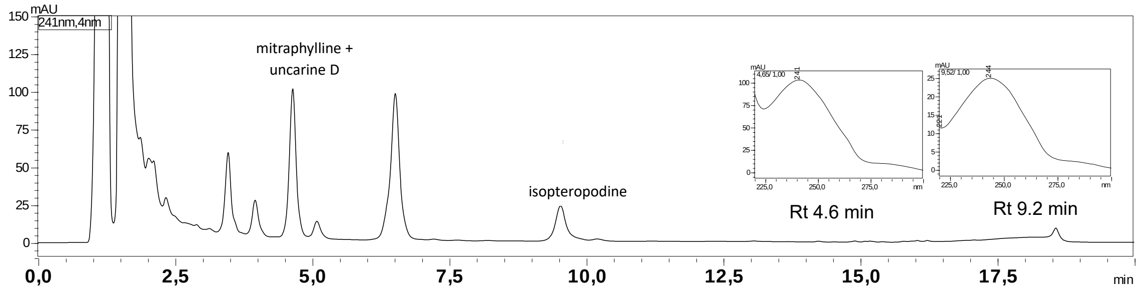

Figure S1. HPLC chromatogram of *Uncaria tomentosa* extract detected at 241 nm, and UV spectra of mitraphylline + uncarin D (Rt = 4.6 min) and isopteropodine (Rt = 9.2 min) peaks.

Table S1. **Calibration curve characteristics, limit of detection and quantification values, and content of alkaloids in the extract.**

	Mitraphylline	Isopteropodine
LoD	7.95 ng/inj	12.78 ng/inj
LoQ	24.08 ng/inj	38.73 ng/inj
Calibration points	10	10
Range covered	0.046 – 2.30 µg/inj	0.054 – 2.72 µg/inj
R^2	0.9999753	0.9999785
Content in 1 g extract	9.430 ± 0.6407 mg	4.265 ± 0.1936 mg

Table S2 – Uptake inhibition transport assay details.

These parameters were set during previous validations. TC: taurocholic acid, E3S: estrone 3-sulfate, CCK-8: cholecystokinin fragment 26-33 amide, MPP^+: 1-Methyl-4-phenylpyridinium, TEA: tetraethylammonium, AMG: Methyl α-D-glucopyranoside

Parental cell	Transporter	Incubation time (min)	Substrate	Substrate concentration (µM)	Incubation temperature (°C)
HEK293	OATP1B1	2	E3S	0.1	37
	OATP1B3	2	CCK-8	1	
	OATP2B1	2	E3S	1	
	OATP1A2	1	E3S	1	
	OAT1	2	Tenofovir	5	
	OAT2	15	Uric acid	25	
	OAT3	3	E3S	1	
	OCT1	5	Metformin	10	
	OCT2	1	Metformin	10	
	OCT3	3	MPP^+	0.02	
	OCTN1	8	TEA	10	
	NTCP	2	TC	2	
	MATE1	15	Metformin	10	
	MATE2K	5	Metformin	10	
MDCKII	ENT1	1	Uridine	1	25
	ENT2	2	Adenosine	1	37
HEK293	ENT4	2	MPP^+	10	
	SGLT2	10	AMG	1	
MDCKII	URAT1	10	Uric acid	20	
HEK293	ASCT1	1	Serine	1	25
	ASCT2	1	Serine	1	
	LAT1	1	Leucine	1	37
	LAT2	1	Leucine	1	
	THTR1	3	Thiamine	0.025	
	THTR2	3	Thiamine	0.025	

Table S3 – Vesicular transport inhibition assay details.

These parameters were set during previous validations. TC: taurocholic acid, E3S: estrone 3-sulfate, NMQ: N-methyl-quinidine, $E_2$17βG: β-Estradiol 17-(β-D-glucuronide), DHEAS: Dehydroepiandrosterone sulfate, CDCF: 5(6)-Carboxy-2',7-dichlorofluorescein B.

Parental cell	Transporter	Incubation time (min)	Substrate	Substrate concentration (µM)	µg membrane/ well	Incubation temperature (°C)
HEK293	BCRP	1	E3S	1	12.5	32
	BSEP	5	TC	0.2	50	37
	MDR1	1	NMQ	1	50	32
	MRP1	5	$E_2$17βG	0.5	50	37
	MRP2	5	$E_2$17βG	100	50	
	MRP3	10	$E_2$17βG	10	50	
	MRP4	1.5	DHEAS	0.5	50	32
	MRP5	5	CDCF	10	50	37

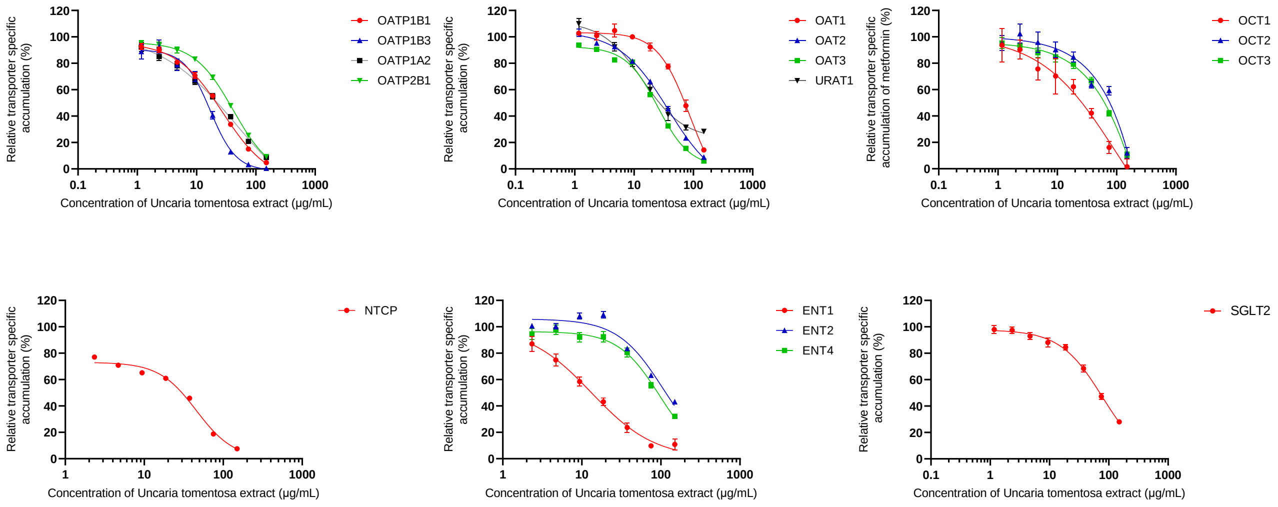

Figure S2 – **Uptake transport inhibition assays.** Dose–response curves of the selected SLC transporters based on decreased activity in the presence of UT extract.**.**
Inhibition of uptake transport with increasing concentrations of UT extract. Cells were preincubated for 15 minutes with the UT extract, then incubated in the presence of UT extract according to the assay parameters listed in the Table S2. Values are shown relative to the vehicle control. All data are presented as the mean ± SEM. The calculated IC_{50} values are shown in the main text.

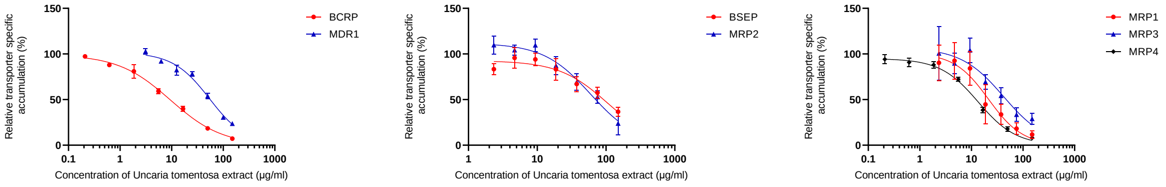

Figure S3 – Vesicular transport inhibition assays. Dose–response curves of the selected membrane vesicles containing ABC transporters based on decreased activity in the presence of UT extract**.** Vesicles were incubated according to the assay parameters as listed in **Error! Reference source not found.**. Values are shown relative to the vehicle control. All data are presented as the mean ± SEM. The calculated IC_{50} values are shown in the main text.

Table S4 – Antiproliferative properties of *Uncaria tomentosa* extract, three natural products (isopteropodine, mitraphylline, and uncarine D) and the reference agent cisplatin.

Cell line	Cell growth inhibition values at the highest concentration[1] (% ± SEM)				cisplatin IC_{50} (µM) [95% confidence interval]
	Uncaria tomentosa extract	isopteropodine	mitraphylline	uncarine D	
HEK293-OCT1	36.56 ± 1.20	<20[2]	<20	<20	9.14 [7.37–11.34]
HEK293-OAT1	20.03 ± 1.64	<20	<20	<20	5.55 [4.60–6.71]
HEK293-Mock-B	<20	<20	<20	<20	13.33 [11.01–16.13]
HEK293-OATP1B1	<20	22.35 ± 2.37	24.45 ± 2.83	<20	4.76 [3.40–6.65]
HEK293-OATP1B3	42.75 ± 2.72	<20	<20	<20	11.23 [8.59–14.68]
HEK293-OATP2B1	<20	27.04 ± 2.92	<20	21.57 ± 2.31	9.70 [7.97–11.81]
HEK293-OATP1A2	<20	31.79 ± 1.51	21.00 ± 0.45	<20	4.90 [3.71–6.48]
HEK293-OCT2	<20	<20	<20	<20	5.51 [4.47–6.81]
HEK293-OAT3	<20	<20	23.16 ± 2.76	<20	4.40 [3.91–4.96]
HEK293-MDR1	<20	<20	<20	<20	4.61 [3.75–5.68]
HEK293-BCRP	<20	<20	<20	<20	5.14 [3.94–6.71]
HEK293-Mock	<20	<20	<20	<20	19.53 [13.42–28.41]
C33A	26.26 ± 1.21	<20	<20	<20	5.85 [5.37–6.38]
SiHa	<20	27.38 ± 2.72	26.22 ± 1.12	<20	4.29 [3.72–4.95]
HeLa	26.99 ± 0.75	<20	<20	<20	12.14 [10.18–14.46]
A2780	<20	<20	<20	<20	5.27 [4.37–6.35]
MCF7	<20	<20	<20	<20	8.34 [7.40–9.40]
MDA-MB-231	<20	<20	<20	<20	25.99 [20.04–33.70]
T47D	<20	<20	<20	<20	17.48 [14.97–]
NIH/3T3	<20	<20	<20	<20	5.50 [4.76–6.35]

[1]: The highest applied concentration was 90 µg/mL and 30 µM for the extract and the pure natural products, respectively.

[2]: Inhibitions less than 20% compared to the untreated control were regarded as negligible and were not given numerically.

Table S5 – Determining if a compound has the potential to inhibit MDR1 and/or BCRP in vivo. Based on ICH M12 guideline recommendations for assessing a compound as a BCRP or MDR1 inhibitor, I_{gut}/IC_{50} ratios were determined, where I_{gut} is the maximum theoretical intestinal concentration (dose/250 mL). Calculations reflect a 500 mg dose of UT extract. A compound has the potential to inhibit MDR1 or BCRP in vivo if it is administered orally, and the I_{gut} /IC_{50} or K_i ≥10.

Transporter	I_{gut}/IC_{50} ratios		
	**Extract**	**Mitraphylline + Uncarine D**	**Isopteropodine**
**BCRP**	188.1	-	0.42
**MDR1**	36.66	1.81	0.82

Table S6 – Localization of the transporters studied, examples of both their drug substrates and known herbal inhibitors and herbal product interactors

Transporter (gene) name	Localization	Drug substrates	Herbal inhibitors	Herbal product	References
OATP1A2 (SLCO1A2)	Brain, intestine, liver kidney, testes, lung, eye	Fexofenadine, ouabain, imatinib, erythromycin, levofloxacin, pitavastatin, pravastatin, rosuvastatin, methotrexate, saquinavir	Naringenin, hesperidin, quercetin, kaempferol, epicatechin gallate (ECG), epigallocatechin gallate (EGCG)	Grapefriuit juice, apple juice, green tea	[1] [2][82][84][85][86] [87] [88]
OATP2B1 (SLCO2B1)	Small intestine, lung, liver, placenta, heart, brain, kidney, skeletal muscle, pancreas	Atorvastatin, Bosentan, Fluvastatin, Glibenclamide, Pravastatin, Rosuvastatin	Naringenin, hesperidin, scutellarin, baicalin glycyrrhizic acid, β-PGG; rutin, icariin, quercetin, kaempferol, theaflavin, EGCG, ECG, ursolic acid, murbellin	Grapefruit juice, rhei rhizoma, perillae herba extract, horny goat weed, black tea, green tea, pomegrante, white mullberry	[89] [83] [90] [91] [88]
OATP1B1 (SLCO1B1)	Liver	Valsartan, atorvastatin, Olmesartan, bosentan, enalapril, fexofenadine, methotrexate, rifampicin, troglitazone sulfate, Fluvastatin, pitavastatin, pravastatin, rosuvastatin	Astragaloside, ginsenoside Rc, 2-O-galloyl hyperin, epimedin C, scutellarin, phlorizin, quercetin, rutin, Icariin, ECG, EGCG, ursolic acid, oleanolic acid, biochanin A, red clover in soy, peanuts ,chickpea	Saussurea Radix, curcumae rhizoma, tomato, buckwheat, horny goat weed, panax ginseng, herba epimedii, pyrola incarnate fisch, breviscapine, apple juice, pomegranate, green tea	[92][93] [88]
OATP1B3 (SLCO1B3)		Valsartan, telmisartan,	Baicalin, baicalein, icariin,	Chinese skullcap, green tea,	[94][83] [88]

		docetaxel, bosentan, fexofenadine, enalapril, fluvastatin, methotrexate, olmesartan, paclitaxel, pitavastatin, rifampicin, rosuvastatin	ursolic acid, gallic acid, betulinic acid	pomegranate, horny goat weed, apple peel, olives	
OAT1 (SLC22A6)	Kidney	Tetracycline, acyclovir, zidovudine, cimetidine, ranitidine, furosemide, ibuprofen, indomethacin	Wedelolactone, wogonin, baicalein, luteolin, quercetin, viscidulin III, 18-β glycyrrhetinic acid, aristolochic acid, morin, silybin, gallic acid	-	[95] [96][97][98]
OAT2 (SLCO22A7)	Liver, corneal epithelium, cancer tissues	Erythromycin, zidovudine, cimetidine, ranitidine, 5-fluorouracil, methotrexate	-	-	[97] [98]
OAT3 (SLCO22A8)	kidney	Ibuprofen, indomethacin, ketoprofen methotrexate, famotidine, cimetidine pravastatin	Dioscorealide B, wedelolactone, oroxylin A, wogonin, luteolin, quercetin, viscidulin III, scullcapflavone II, aristolochic acid, gallic acid	-	[95] [96] [97] [98]
OCT1 (SLC22A1)	Liver, intestine, neurons	Prazosin, verapamil, amoxicillin, lamotrigine, salbutamol, metformin	Nuciferine, berberine, retrorsine, anisodine, monocrotaline, quercetin	-	[99] [97] [100]
OCT2 (SLC22A2)	Kidney, neurons	Cisplatin, metformin, amiloride, ranitidine, ethambutol, sumatriptan	Beriberine, epigallocatechin gallate, epicatechin-3-gallate	Rhizoma Coptidis, green tea	[101][100][102]
OCT3 (SLC22A3)	Liver, placenta, kidney, intestine	Metformin, oxaliplatin, ethidium, prazosin,	Wogonin	-	[96][103][104]
OCTN1 (SLC22A4)	Kidney, intestine, heart, spleen, brain, lung,	Amisulpiride, ethambutol, 5-Fluorouracil, gabapentin, metformin,	L-ergothioneine	Mushrooms	[11][105][106]

	skeletal muscle	cytarabine, ribavirin, verapamil, tiotropium			
URAT1 (SLC22A12)	Kidney	-	Baicalein, naringenin, hesperetin, nobiletin	Citrus fruits	[107][108][109]
MATE1 (SLC47A1)	Kidney, liver, skeletal muscle, adrenal gland, placenta	Metformin, cimetidine, procainamide, guanidine, acyclovir, cephalexin	Berberine, epigallocatechin gallate	Tree turmeric, barberry	[110][111][112][102]
MATE2K (SLC47A2)	kidney	Metformin, cimetidine, procainamide, guanidine, acyclovir	Epigallocatechin gallate	Green tea	[110] [112][102]
ENT1 (SLC29A1)	Ubiquitous	Citarabine, cladribine, zalcitabine, rivabirine, dipyridamole	Curcumin, cannabidiol	-	[113][114][115][116]
ENT2 (SLC29A2)	ubiquitous	Fludarabine, cladribine, zidovudine, dipyridamole	-	-	[113][114]
ENT4 (SLC29A4)	brain, small intestine, heart, kidney	metformin	-	-	[117]
NTCP (SLC10A1)	liver	rosuvastatin, pitavastatin, fluvastatin, atorvastatin	Ginsenoside Rg1, ophiopogon D', schizandrin A	Shengmai Formula	[118][119]
LAT1 (SLC7A5)	Ubiquitous (brain, placenta tumours)	Melphalan, baclofen, gabapentin, pregabalin	-	-	[120][121]
LAT2 (SLC7A8)	Ubiquitous (kidney, intestine)	Doxorubicin	-	-	[122][121]
THTR1 (SLC19A2)	Intestine, liver, erythrocytes, BBB, kidney, placenta, muscle	-	-	-	[123][124]
THTR2 (SLC19A3)		Metformin, famotidine	-	-	[123][124]
ASCT1 (SLC1A4)	Ubiquitous (skeletal muscle, lung, kidney, ovaries, heart, brain)	-	-	-	[125]

ASCT2 (SLC1A5)	Ubiquitous (skeletal muscle, lung, intestine, kidney, testis, brain, T cells)	-	-	-	[125]
SGLT2 (SLC5A2)	Kidney, pancreas	-	-	Polyherbal formulation of: *Artemisa roxburghiana, Cissampelos pareira L., Stephania glabra, Drimia indica, Roylea cinerea, Tinospora sinensis* and *Curcuma longa L.*	[126][127][128]
BSEP (ABCB11)	Liver		Saikosaponin b1, b2, saikogenin A and D, diosbulbin B, kansuinin A, atractyloside, celastrol, obtusin and kansuinin B	Chinese Herb Medicine	[129][130]
BCRP (ABCG2)	Intestine, liver, kidney, brain, placenta, stem cells, mammary glands	Mitoxantrone, topotecan, methotrexate, imatinib, gefitinib, cimetidine, sulfasalazine, pantoprazole, amisulpride, ranitidine	Rutin, resveratrol, apigenin, Berberine, caffein, curcumin, naringenin, baicalin	*M. chamomilla* (Chamomile) *M. officinalis* (Lemon balm) *P. emblica* (Emblic myrobalan) *S. costus* (Costus), *C. chinensis* (Chinese goldthread) *C. japonica*	[111][131] [132][133][134][135]
MDR1 (ABCB1)	Intestine, kidney, liver, brain, placenta	Verapamil, colhicine, daunorubicin, doxorubicin, paclitaxel, amisulpride, saquinavir	Bajijiasu, quercetin, apigenin, α-asarone, β-asarone, avenanthramide (A, B, C), berberine, bilobalide, capsaicin, curcumin, gingerol, naringenin, quercetin, baicalin	*M. chamomilla* (Chamomile) *M. officinalis* (Lemon balm) *P. emblica* (Emblic myrobalan) *S. costus* (Costus), *A. sativa* (Oat), *C. chinensis* (Chinese goldthread) *C. japonica* (Chamelia), *G. biloba* (Gingko), chili peppers, grapefruit juice, green tea	[111][131][132] [133][134] [135]
MRP1 (ABCC1)	testis, cardiomyocytes,	Methotrexate, doxorubicin, etoposide,	Bisbenzylisoquinoline, licoisoflavone A,	-	[136][137][138][139]

	placenta, prostate, lung, thymus, kidney, BBB, smooth muscle cells and endothelial cells of the heart	vincristine, paclitaxel, saquinavir, ritonavir, difloxacin	euchrestaflavanone A, apigenin, naringenin, sophoraflavanone B		
MRP2 (ABCC2)	Liver, intestine, kidney	Methotrexate, estrone 3-sulfate, doxorubicin, epirubicin, ampicillin, azithromycin, cefodizime, olmesartan	Epiifedelanol, resveratrol, baicalin	Rhizoma, grapes	[140][102][141][142] [135]
MRP3 (ABCC3)	Intestine pancreas, gallbladder, liver	Etoposide, doxorubicin, vincristine, cisplatin, methotrexate	Resveratrol, baicalin	-	[111] [135][143]
MRP4 (ABCC4)	Kidney, prostate, liver, testis, ovary, lung neurons, blood cells	Methotrexate, ceftizoxime, cefazolin, cefmetazole, olmesartan, edaravone glucuronide	baicalin	-	[135][144][145][146][147]
MRP5 (ABCC5)	Brain, skeletal muscle, lung, heart	Gemcitabine, cytarabine, methotrexate, fluorouracil, rosuvastatin, atorvastatin	-	-	[148][149][150]

Table S7 – Transporter interactions of active substances of UT described in the literature.

Compound	PubChem CID	Transporter	Type of interaction	Ref
Rhynchophylline	409518	MDR1	no substrate	[151]
			substrate	[152]
Alpha-bisbolol	10586	OATP1B1/3	< 50% inhibition	[75]
alpha-Hederagenin	258538	SERT	IC_{50}: 1.34± 0.04 nM	[153]
		NET	IC_{50}: 0.15± 0.02 nM	
		DAT	IC_{50}: 1.09± 0.12 nM	
5,7-Dihydroxyflavanone	238782	OATP1B1/3	< 50% inhibition	[75]

Abietic acid	90895	OATP1B1/3	Ki: 1.47/ 4.08 µM	[75]
Pinosylvin 3-(methyl ether)	182229	OATP1B1/3	< 50% inhibition	[75]
Stigmast-5-en-3beta-ol	86821	OATP1B1/3	< 50% inhibition	[75]
Beta-Sitosterol	86821	MDR1	no substrate, no inhibitor	[154]
		mouse MRP1		
		Rat BSEP		
		mBCRP		
		ABCG5/8	probably substrate	[155]
Borneol	64685	Rat MDR1	inhibitor	[156]
		rNTCP	inhibited the transcription and the expression	[157]
		rMDR1a		
		rMRP2		
		rMRP4		
		rMRP1		[156]
		MDR1	inhibitor	[158]
